# Comparison of bipolar hemiarthroplasty and total hip arthroplasty for displaced femoral neck fractures in the healthy elderly: a meta-analysis

**DOI:** 10.1186/s12891-015-0696-x

**Published:** 2015-08-28

**Authors:** Feng Wang, Haifei Zhang, Zhiyu Zhang, Chengbin Ma, Ximin Feng

**Affiliations:** Department of Orthopedics, the Fourth Affiliated Hospital of China Medical University, Chongshan Road, Shenyang, 110032 Liaoning People’s Republic of China

## Abstract

**Background:**

Displaced femoral neck fractures (FNFs) in healthy elderly patients have traditionally been managed with hemiarthroplasty (HA) or total hip arthroplasty (THA), with studies suggesting that THA may be the better option. However, it has recently been reported that bipolar HA (BHA) also provides good outcomes, and it is not clear as to whether BHA or THA is most appropriate. The purpose of this study was to conduct a meta-analysis of randomized controlled trials (RCTs) comparing the outcomes of BHA with THA for treating FNF in healthy elderly patients.

**Methods:**

We searched the following databases from inception to May 2015 for relevant RCTs without language restrictions: PubMed, the Cochrane Central Register of Controlled Trials, Ovid MEDLINE and EMBASE, CINAHL, the China Biological Medicine Database, International Clinical Trials Registry Platform, Current Controlled Trials, and ClinicalTrials.gov. RCTs that met the inclusion criteria were statistically analyzed using the Cochrane review methods.

**Results:**

Eight RCTs were included (total 1,014 patients; 523 had BHA and 491 had THA). The data from included RCTs were divided into four subgroups according to different follow-up durations. The Harris Hip Score after BHA was not different from that after THA in all subgroups. Both reoperation rate and acetabular erosion rate were higher after BHA after more than 4 years, while there was a higher dislocation rate associated with THA within 4 years. THA was more favorable regarding the EQ_index_-5D and the mobility and pain rate, while BHA was more favorable regarding operating time. No significant differences were found regarding infection rate, general complications, 1-year mortality, blood loss, and length of postoperative hospital stay.

**Conclusions:**

For healthy elderly patients with displaced FNFs, treatment with BHA led to better outcomes regarding dislocation rate, while THA was better regarding acetabular erosion rate and reoperation rate. When comparing BHA with THA, there were no significant differences in other important outcomes such as Harris Hip Score, infection rate, general complications, and 1-year mortality. Further high-quality RCTs are needed to provide robust evidence and evaluate the treatment options.

## Background

The proportion of elderly people is increasing as the world’s population ages, resulting in an expected rise in the incidence of osteoporotic hip fractures. It is estimated that about 1.6 million hip fractures occurred in the year 2000 [1], and the incidence of hip fractures is expected to increase to over six million worldwide by the year 2050 [2]. About half of the hip fracture population has displaced femoral neck fracture (FNF, Garden type III or IV) of the subcapital region [3]; displaced FNFs can result in non-union or avascular necrosis [4]. Moreover, these fractures are associated with impaired mobility, loss of function, and personal dependence as well as with global economic health costs, and are significant causes of mortality and morbidity in the elderly [5, 6]. The optimal treatment of displaced FNF in the elderly is an ongoing scientific and clinical debate [7].

Surgical treatment options for displaced FNF include internal fixation, which is not recommended in elderly patients [8–11], and arthroplasty. Both hemiarthroplasty (HA) and total hip arthroplasty (THA) are widely accepted methods of hip replacement after displaced FNF. Some evidence has suggested that THA leads to better functional outcome than HA [7]; however, there are some advantages of HA compared with THA such as reduced dislocation rate, less complex surgery, shorter operation time, less blood loss, and lower initial costs [12].

The prosthesis and the acetabulum in HA can be articulated using a unipolar or bipolar prosthesis. Parker et al. reported no difference in outcome between bipolar and unipolar prostheses in adults [7]. However, recent studies have reported that bipolar HA (BHA) provides good outcomes for elderly patients with displaced FNFs. BHA after FNF has predictable and good medium- and long-term results, even when compared with internal fixation or unipolar HA [8, 13, 14], and BHA displays a later onset of acetabular erosion compared with unipolar HA [14, 15]. A review of data from national registries supports the continued use of BHA for FNF in the elderly [16], implying that BHA should be the preferred treatment for elderly patients with displaced FNF. Elderly patients who receive BHA may also have a more favorable survival outcome compared with those who receive unipolar HA [17]. Additionally, in elderly patients with FNFs who were fit and physiologically young, uncemented BHA seemed to achieve better functional outcomes [18]. The clinical results from different groups cannot agree on whether to recommend BHA or THA [19–21].

Therefore, we performed a meta-analysis of randomized controlled trials (RCTs) published up to May 2015. The aim of this study was to evaluate the clinical outcomes of BHA compared with THA. The results will improve understanding of the treatment options for displaced FNFs in healthy elderly patients.

## Methods

### Literature search

A protocol was developed prior to commencement of this meta-analysis following the Cochrane Back Review Group guidelines [22]. We searched the following databases from inception to May 2015 for relevant RCTs without language restrictions: PubMed, the Cochrane Central Register of Controlled Trials (CENTRAL), Ovid MEDLINE and EMBASE, CINAHL, the China Biological Medicine Database (CBM), International Clinical Trials Registry Platform (ICTRP), Current Controlled Trials, and ClinicalTrials.gov. Other search methods included screening references listed in relevant systematic reviews and identified RCTs, searching the abstracts of relevant meetings, and personal communication with content experts in the field and authors of identified RCTs. Key words used for searching were arthroplasty, hip prosthesis implantation, total hip replacement, bipolar hemiarthroplasty, hip fractures, femoral neck fracture, and randomized controlled trial.

### Study eligibility criteria

All RCTs comparing BHA with THA for the treatment of FNF were considered for this review. Trials including participants with displaced FNFs (Garden type III or IV) in the elderly (older than 65 years) were eligible. Patients with the following conditions were excluded from the study: (1) undisplaced FNF; (2) pathological fracture secondary to malignant disease; (3) osteoarthritis or rheumatoid arthritis of the hip; (4) severe cognitive dysfunction; or (5) non-first time experience with artificial joint replacement.

The first phase of the trial selection process involved screening of titles and abstracts, followed by a second phase of eligibility evaluation from the full-text format. Both phases were performed independently by two reviewers and checked by the principal reviewer. The observed percentage agreement between the reviewers for the assessment of inclusion was calculated using the κ test [23, 24]. Disagreements were resolved by discussion.

### Risk of bias assessment and evaluation of validity

The risk of bias (RoB) and methodological quality was assessed in duplicate using the Cochrane Collaboration recommendations, and evaluated independently by two reviewers [22, 25]. The criteria included six items as follows: (1) adequate sequence generation; (2) allocation concealment; (3) blinding; (4) incomplete outcome data; (5) selective reporting; and (6) other bias.

### Data extraction

The data were extracted from included reports independently by two reviewers, and any disagreements were resolved through discussion. The data extracted included: participant characteristics, number of participants, and loss to follow-up; study characteristics; intervention details; primary and secondary outcomes. The primary outcomes included the Harris Hip Score (HHS) and reoperation rate. The secondary outcomes included: mobility, dislocation, acetabular erosion, infection, general complications, 1-year mortality, EuroQol (EQ)_index_-5D score, pain rate, operation time and blood loss, and length of hospital stay.

### Assessment of heterogeneity

Heterogeneity was informally tested visually by the eyeball test, and formally tested by the chi-squared test and the I^2^ statistic; however, the decision regarding heterogeneity was dependent on I^2^. Substantial heterogeneity was defined as ≥50 %, and the effect of the interventions was described if the results were too heterogeneous.

### Measures of treatment effect

Attempts were made to statistically pool the data of homogeneous studies in order to obtain the primary and secondary outcomes. The results were expressed in terms of risk ratio (RR) and 95 % confidence intervals (95 % CI) for dichotomous outcomes, and in terms of weighted mean difference and 95 % CI for continuous outcomes. When the same continuous outcomes were measured by different scales, the standardized mean difference and 95 % CI were calculated. The individual and pooled statistics were calculated using the random effect model. Funnel plots were used to explore potential publication bias. The Cochrane Review Manager software (RevMan version 5.1, the Nordic Cochrane Centre, København, Denmark) software was used for data analysis.

## Results

### Search results

**Fig. 1:**
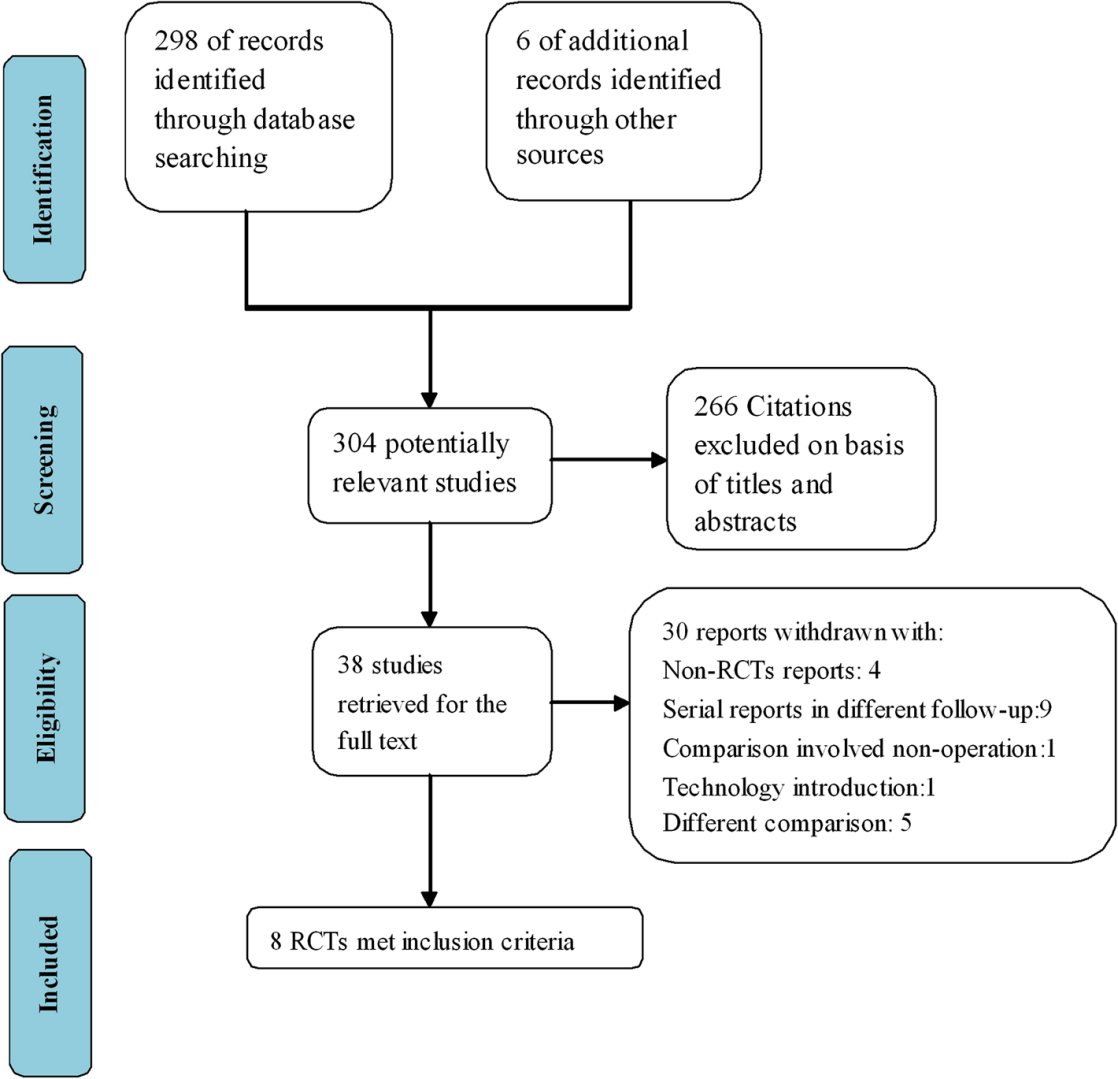
PRISMA flow diagram of study selection

The primary search identified 304 records, with 266 publications immediately excluded based on title and abstract screening. From the remaining 38 publications identified as potentially relevant, 30 were omitted according to the inclusion and exclusion criteria. A final total of eight trials [12, 19, 26–31] were included in the meta-analysis (Fig. 1). The κ statistic for interrater agreement regarding study eligibility was 0.81.

The eight RCTs eligible for inclusion involved a total of 1,014 patients, with individual sample sizes ranging from 81 to 252 patients. All studies in our meta-analysis were written in English, and were published between 1986 and 2013. The follow-up period ranged from 12 months to 13 years. Each included RCT presented baseline demographic data such as age, sex, and race. The characteristics of these studies are presented in Table 1.

**Table 1:** Overview of included trials

1st Author, publication year	Country conducted	Preoperative diagnosis	Comparisons	Sample size T/C	Female (%)	Mean age(year) T/C	Follow-up (month)
Dorr LD 1986	United States	Displaced FNF	THA vs. BHA	39/50	65.17	69/69	24–48
Ravikumar 2000	United Kingdom	Displaced FNF	THA vs. BHA	89/91	90	81.03/82.06	>48
Keating 2005	United Kingdom	Displaced FNF	THA vs. BHA	69/69	76.8	75.2/75.0	24
Baker 2006	United Kingdom	Displaced FNF	THA vs. BHA	40/41	79.01	74.2/75.83	30–68
Mouzopoulos 2008	Greece	Displaced FNF	THA vs. BHA	37/34	73.23	73.07/74.24	48
van den Bekerom 2010	Netherlands	Displaced FNF	THA vs. BHA	115/137	81	80.3/82.1	>48
Hedbeck 2011	Sweden	Displaced FNF	THA vs. BHA	60/60	84.17	80.5/80.7	48
Cadossi,2013	Italy	Displaced FNF	THA vs. BHA	42/41	74.7	82.3/84.2	36

### Methodological quality of included studies

**Fig. 2:**
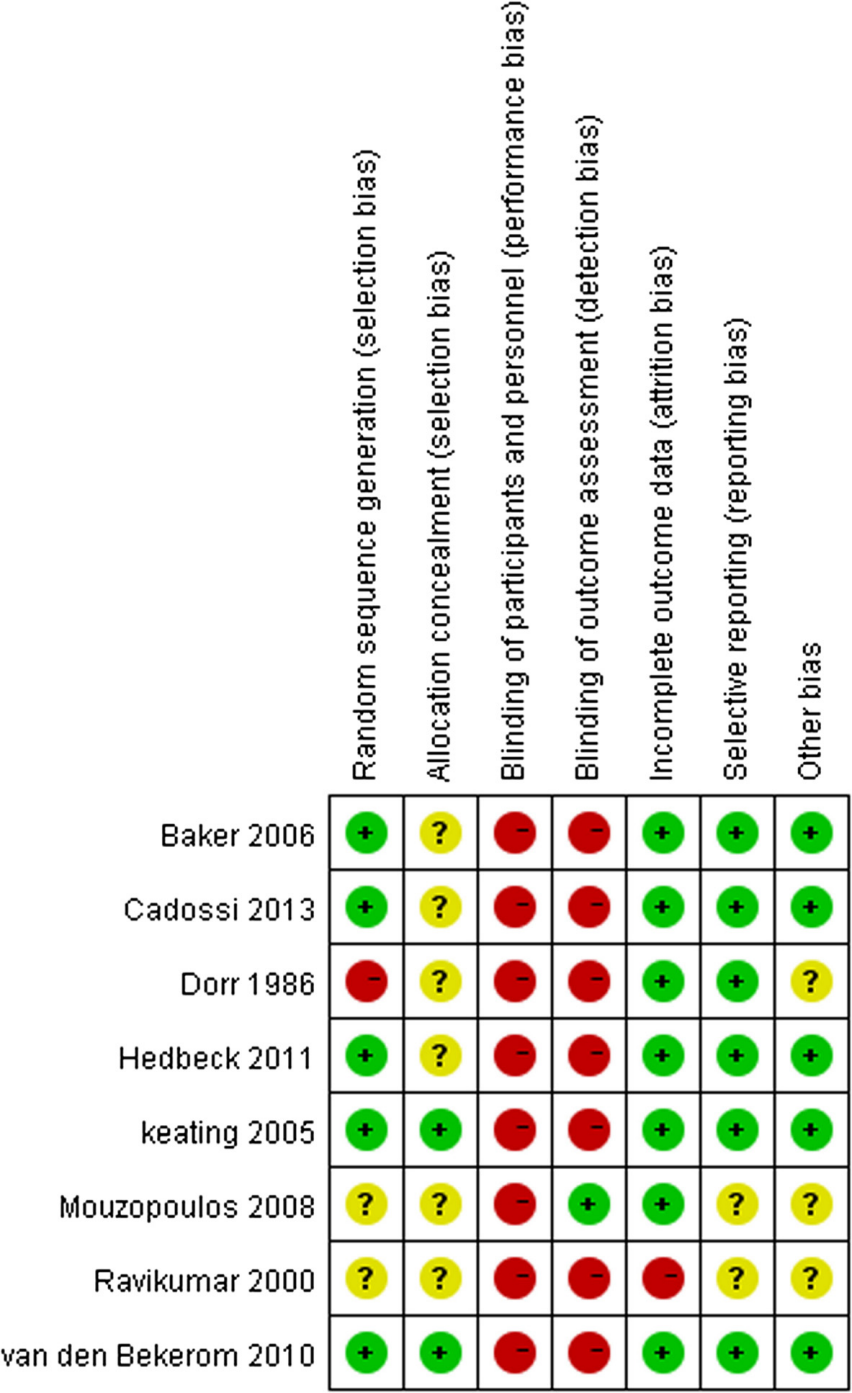
Risk of bias assessment summary of included studies

The results of the RoB assessment for the included studies are summarized in Fig. 2. Five studies had adequate methods of randomization [12, 19, 28, 30, 31], and two studies used an adequate sequence generation and allocation procedure [12, 19]. Both randomization and allocation were unclear in two included studies [27, 29]. None of the included studies attempted to blind the patients or surgeon as this was impossible owing to the nature of the surgery; one study compensated for the lack of blinding by using blinded observers to assess the outcome [29]. Most of the included studies provided an adequate overview of withdrawals or dropouts, and were able to keep these to a minimum for the subsequent follow-up measurements, although only two studies conducted long-term follow-up [19, 27]. Published or registered protocols were unavailable for all studies, even after we conducted a comprehensive search. In the absence of these, it was difficult for us to decide whether outcomes were measured, or not reported because they were found to be insignificant or unfavorable. Therefore, six included studies that reported most primary outcomes were considered to have fulfilled this criterion [12, 19, 26, 28, 30, 31].

### Quantitative data synthesis of the primary outcomes

#### Harris hip score

**Fig. 3:**
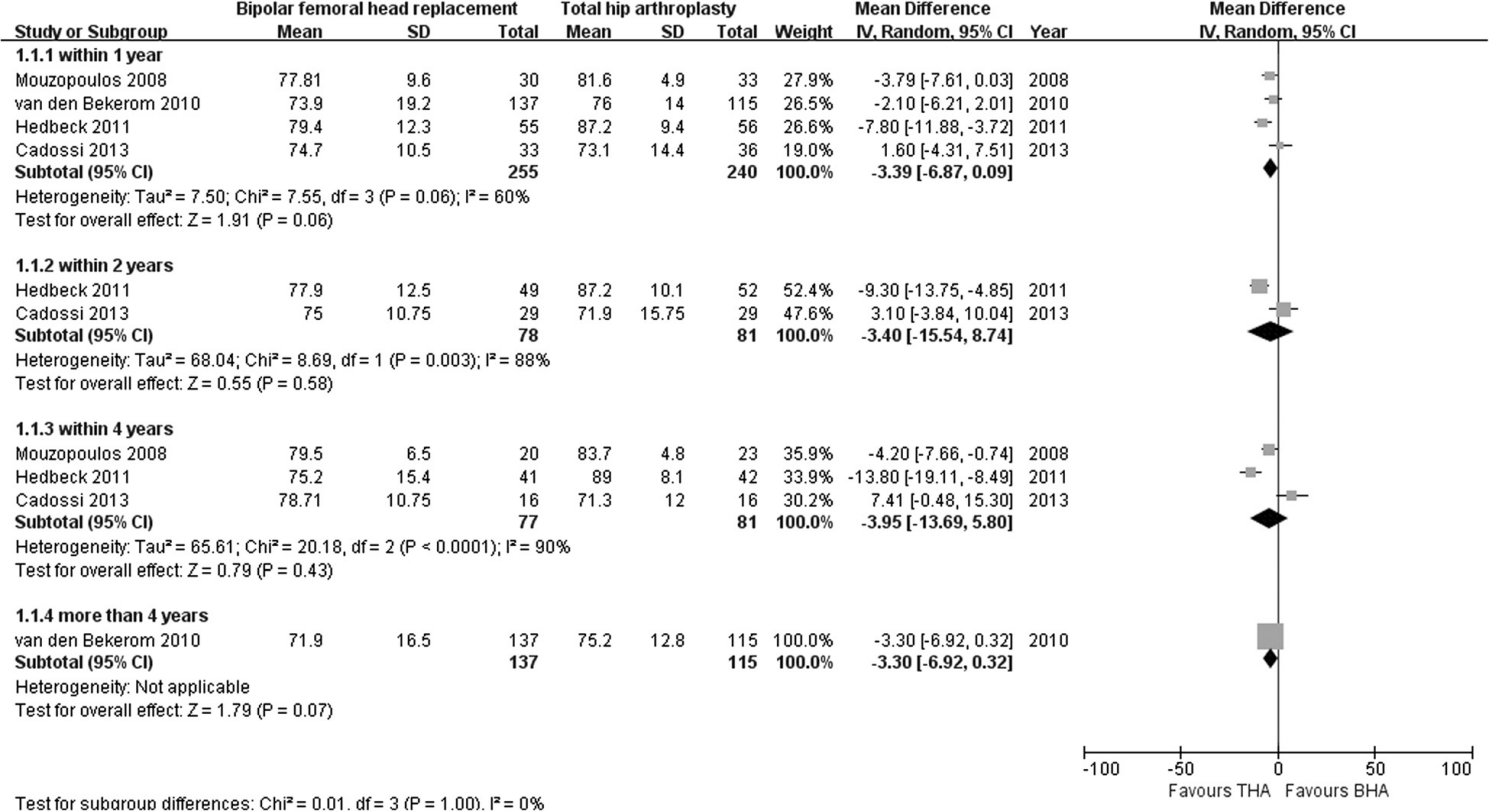
Forest plot of Harris hip scores after bipolar hemiarthroplasty compared with those after total hip arthroplasty

The HHS is frequently used to assess the clinical outcome of total hip replacement. We selected it as the primary outcome owing to its high validity and reliability. Four studies assessed the HHS after BHA compared with THA within 1 year of follow-up (255 with BHA versus 240 with THA) [19, 29–31]; no significant differences were found (MD −3.39; 95 % CI −6.87, 0.09; *p* = 0.06), and heterogeneity across the studies was obvious (I^2^ = 60 %). Two studies assessed the HHS after BHA compared with THA within 2 years of follow-up (78 with BHA versus 81 with THA) [30, 31]; again no significant differences were found (MD −3.40; 95 % CI −15.54, 8.74; *p* = 0.58), and heterogeneity across the studies was obvious (I^2^ = 88 %). Three studies assessed the HHS after BHA compared with THA within 4 years of follow-up (77 with BHA versus 81 with THA) [29–31]; no significant differences were found (MD 3.95; 95 % CI −5.80, 13.69; *p* = 0.43), and heterogeneity across the studies was obvious (I^2^ = 90 %). Only one study reported no significant differences in HHS after BHA compared with THA after 5 years of follow-up (MD 3.30; 95 % CI −0.32, 6.92; *p* = 0.07; Fig. 3) [19].

#### Reoperation rate

**Fig. 4:**
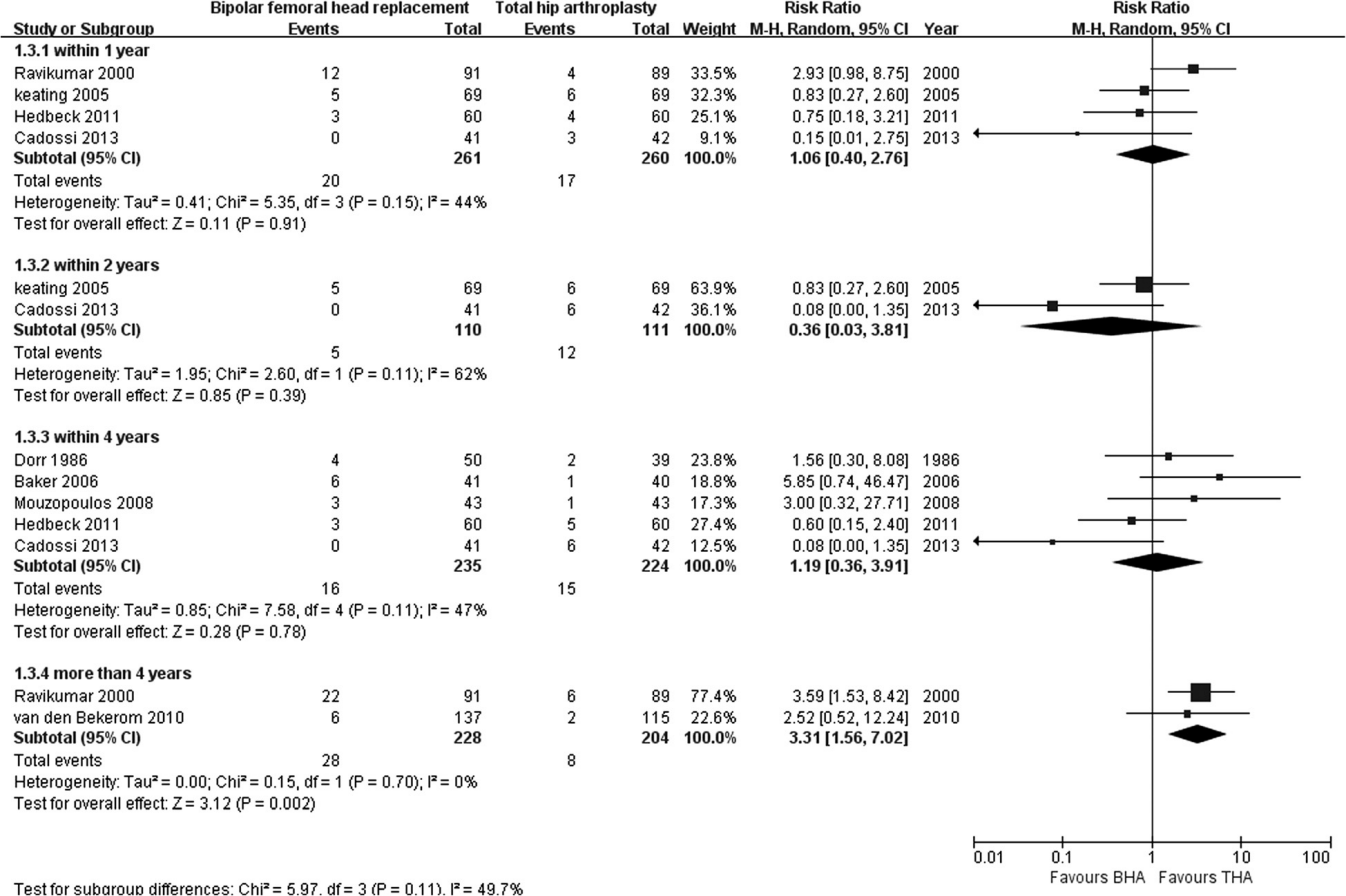
Forest plot of reoperation rate after bipolar hemiarthroplasty compared with that after total hip arthroplasty

Four studies assessed the reoperation rate after BHA compared with THA within 1 year of follow-up (261 with BHA versus 260 with THA) [12, 27, 30, 31]; no significant differences were found (RR 1.06; 95 % CI 0.40–2.76; *p* = 0.91), and there was no heterogeneity across the studies (I^2^ = 44 %). Two studies assessed the reoperation rate after BHA compared with THA within 2 years of follow-up (110 with BHA versus 111 with THA) [12, 31]; no significant differences were found (RR 0.36; 95 % CI 0.03–3.81; *p* = 0.39),but heterogeneity across the studies was obvious (I^2^ = 62 %). Five studies assessed the reoperation rate after BHA compared with THA within 4 years of follow-up (235 with BHA versus 224 with THA) [26, 28–31]; no significant differences were found (RR 1.19; 95 % CI 0.36–3.91; *p* = 0.78), and there was no heterogeneity across the studies (I^2^ = 47 %). Two studies reported that the reoperation rate after BHA was significantly greater than that after THA with more than 4 years of follow-up (228 with BHA versus 204 with THA; RR 3.31; 95 % CI 1.56–7.02; *p* = 0.002; I^2^ = 49.7 %; Fig. 4) [19, 27].

### Quantitative data synthesis of the secondary outcomes

#### Mobility

**Fig. 5:**
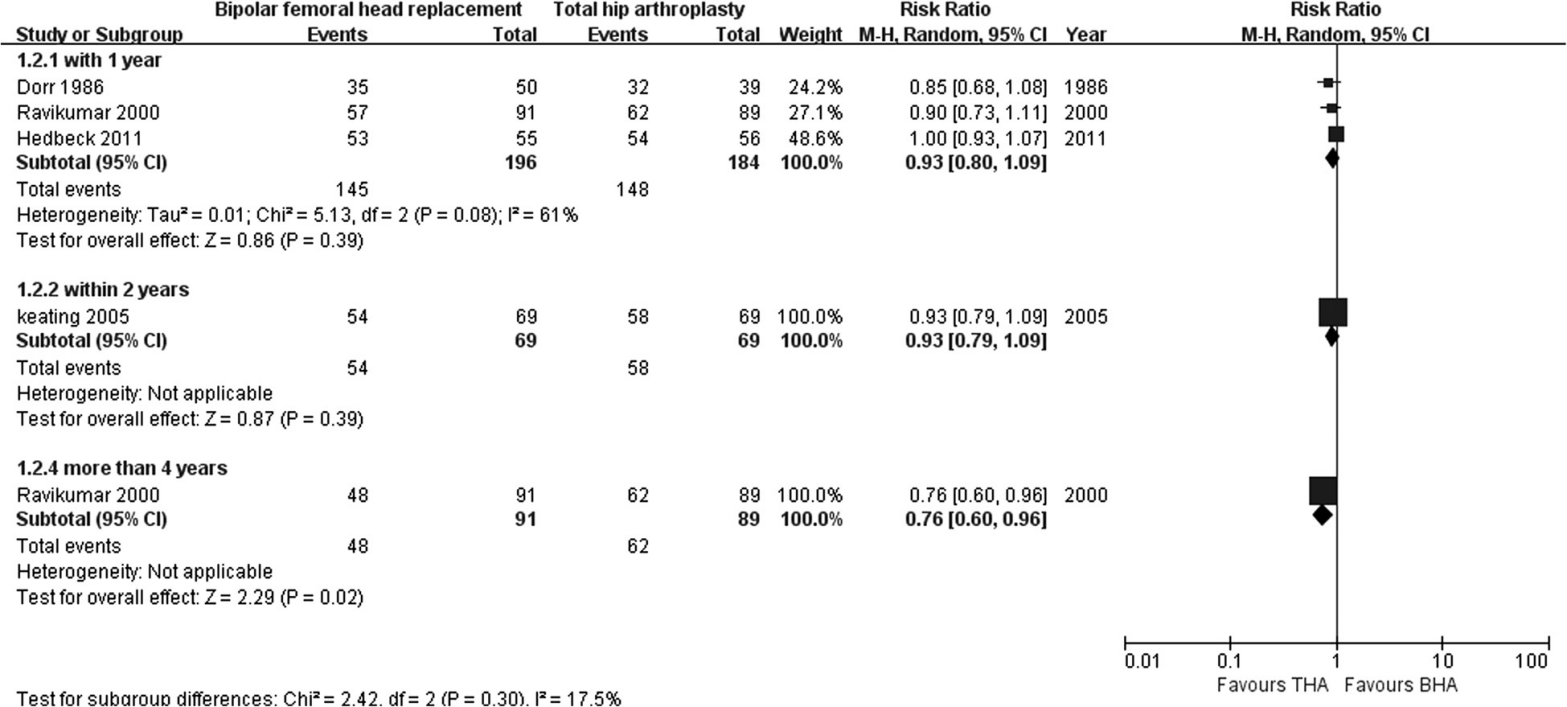
Forest plot of mobility after bipolar hemiarthroplasty compared with that after total hip arthroplasty

Three studies assessed the mobility rate after BHA compared with that after THA within 1 year of follow-up (196 with BHA versus 184 with THA) [26, 27, 30]; no significant differences were found (RR 0.93; 95 % CI 0.80–1.09; *p* = 0.39), and heterogeneity across the studies was obvious (I^2^ = 61 %). One study showed similar results within 2 years of follow-up (RR 0.93; 95 % CI 0.79–1.09; *p* = 0 .39) [12]. However, another study reported significantly higher mobility rates after THA compared with BHA within 5 years of follow-up (RR 0.76; 95 % CI 0.60–0.96; *p* = 0.02; Fig. 5) [27].

#### Dislocation rate

**Fig. 6:**
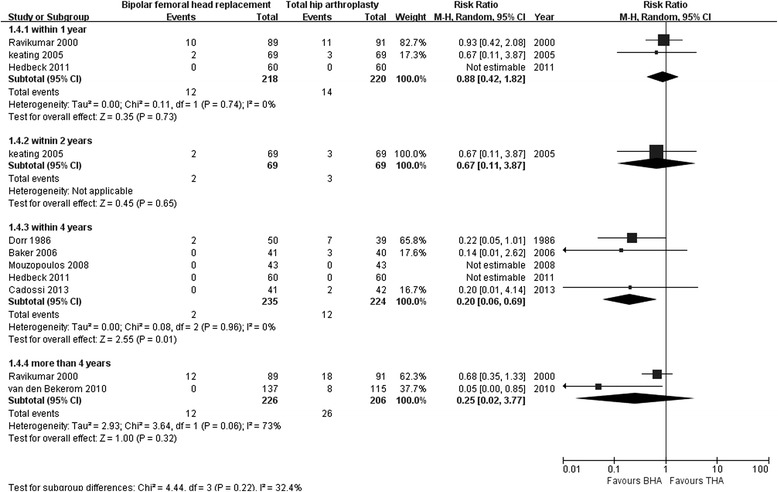
Forest plot of dislocation rate after bipolar hemiarthroplasty compared with that after total hip arthroplasty

Three studies assessed the dislocation rate after BHA compared with THA within 1 year of follow-up (218 with BHA versus 220 with THA) [12, 27, 30]; no significant differences were found (RR 0.88; 95 % CI 0.42–1.82; *p* = 0.73), and there was no heterogeneity (I^2^ = 0 %). Only one study showed similar results for the dislocation rate after both procedures within 2 years of follow-up (RR 0.67; 95 % CI 0.11–3.87; *p* = 0.65) [12]. Five studies reported a significantly greater dislocation rate after THA compared with BHA within 4 years of follow-up (RR 0.20; 95 % CI 0.06–0.69; *p* = 0 .01), and there was no substantial heterogeneity between studies (I^2^ = 0 %) [26, 28–31]. Two studies reported no significant difference in dislocation rate after BHA compared with that after THA after more than 4 years of follow-up (226 with BHA versus 206 with THA; RR 0.25; 95 % CI 0.02–3.37; *p* = 0.32), and heterogeneity across the studies was obvious (I^2^ = 73 %; Fig. 6) [19, 27].

#### Acetabular erosion rate

**Fig. 7:**
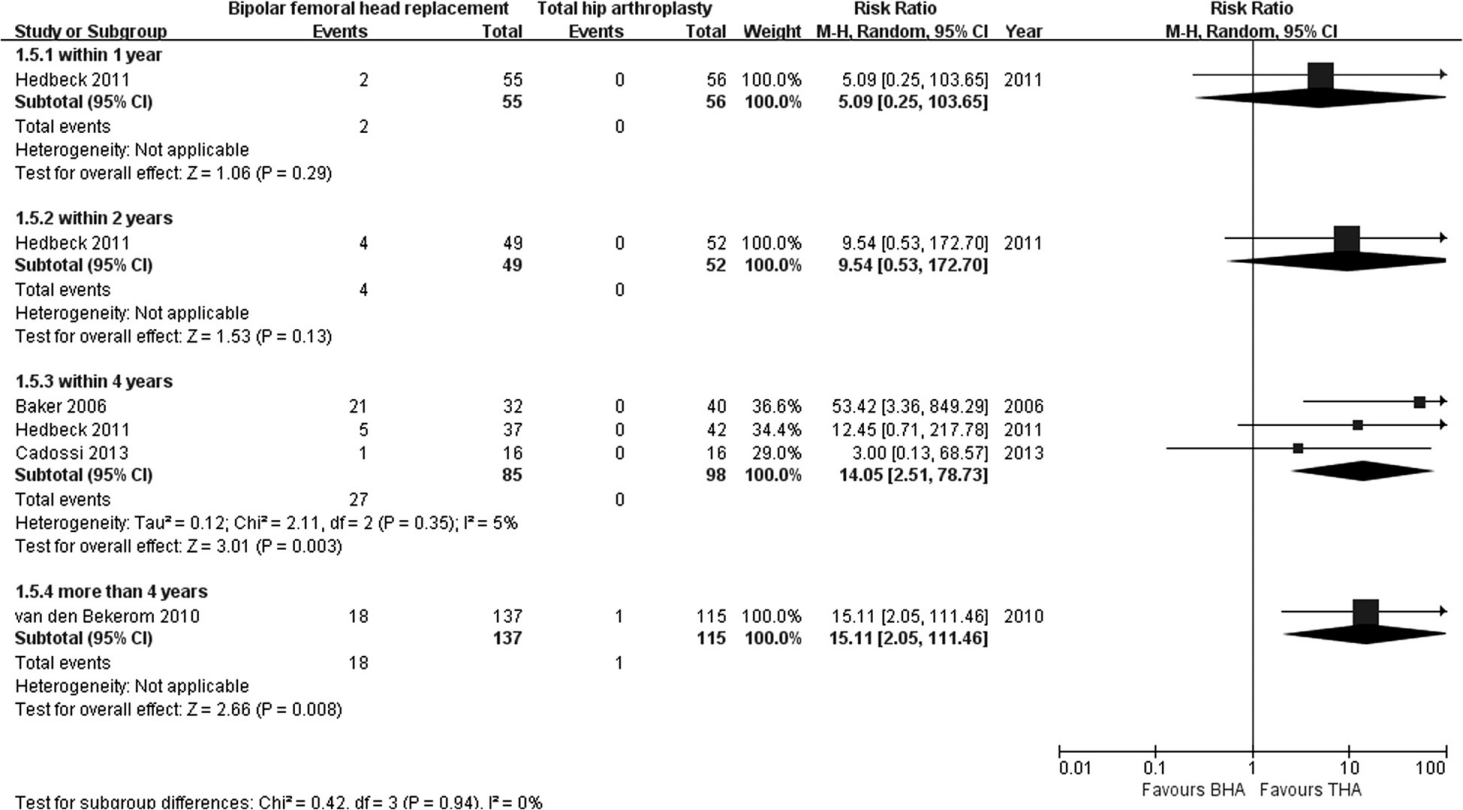
Forest plot of acetabular erosion rate after bipolar hemiarthroplasty compared with that after total hip arthroplasty

One study reported no significant differences in acetabular erosion rate after BHA compared with that after THA within 1 year of follow-up (55 with BHA versus 56 with THA; RR 5.09; 95 % CI 0.25–103.65; *p* = 0.29), and similar results were reported after 2 years of follow-up (RR 9.54; 95 % CI 0.53–172.70; *p* = 0.13) [30]. Three studies reported significantly higher acetabular erosion rates after BHA compared with that after THA within 4 years of follow-up (85 with BHA versus 98 with THA; RR 14.05; 95 % CI 2.51–78.73; *p* = 0.003), and heterogeneity was minor (I^2^ = 5 %) [28, 30, 31]. One study also reported significantly higher acetabular erosion rates after BHA compared with that after THA within 5 years of follow-up (RR 15.11; 95 % CI 2.05–111.46; *p* = 0.008; Fig. 7) [19].

#### Infection

**Fig. 8:**
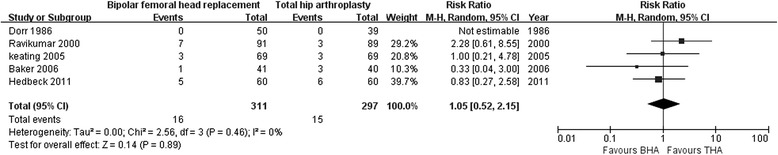
Forest plot of infection rate after bipolar hemiarthroplasty compared with that after total hip arthroplasty

The infection rate of the BHA group and the THA group was available in five studies (311 with BHA versus 297 with THA) [12, 26–28, 30]; no significant difference in infection rate was found between the two groups (RR 1.05, 95 % CI 0.52–2.15; *p* = 0.89), and there was no substantial heterogeneity (I^2^ = 0 %; Fig. 8).

#### General complications

**Fig. 9:**
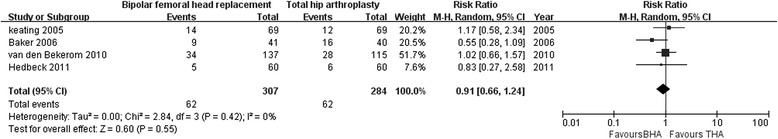
Forest plot of general complication rate after bipolar hemiarthroplasty compared with that after total hip arthroplasty

Four studies assessed the general complication rate associated with BHA compared with that associated with THA (307 with BHA versus 284 with THA) [12, 19, 28, 30]; no significant differences were found (RR 0.91; 95 % CI 0.66–1.24; *p* = 0.55), and there was no substantial heterogeneity (I^2^ = 0 %; Fig. 9).

#### One-year mortality

**Fig. 10:**
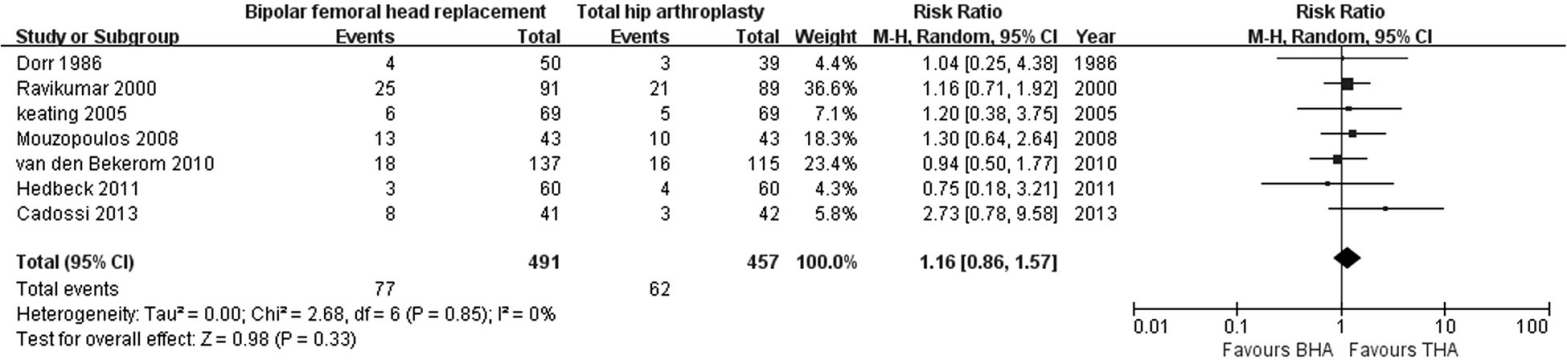
Forest plot of 1-year mortality after bipolar hemiarthroplasty compared with that after total hip arthroplasty

Seven studies assessed the 1-year mortality rate after BHA compared with that after THA (491 with BHA versus 457 with THA) [12, 19, 26, 27, 29–31]; no significant differences were found (RR 1.16; 95 % CI 0.86–1.57; *p* = 0.33), and there was no substantial heterogeneity (I^2^ = 0 %; Fig. 10).

#### Others

**Fig. 11:**
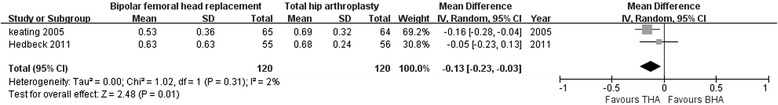
Forest plot of EQ-5D scores after bipolar hemiarthroplasty compared with that after total hip arthroplasty

**Fig. 12:**
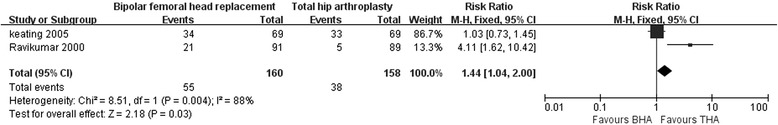
Forest plot of pain rate after bipolar hemiarthroplasty compared with that after total hip arthroplasty

**Fig. 13:**
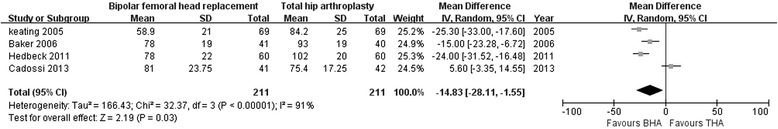
Forest plot of operating time for bipolar hemiarthroplasty compared with that for total hip arthroplasty

**Fig. 14:**
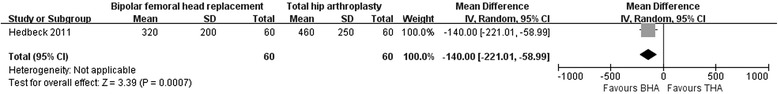
Forest plot of blood loss during bipolar hemiarthroplasty compared with that during total hip arthroplasty

**Fig. 15:**
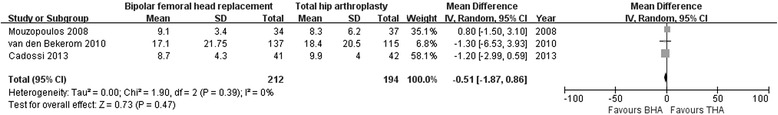
Forest plot of length of hospital stay after bipolar hemiarthroplasty compared with that after total hip arthroplasty

EQ_index_-5D scores were available from two included studies [12, 30]. The pooled result showed a significant difference between the two procedures (MD −0.13; 95 % CI −0.23, 0.13; *p* = 0.01; Fig. 11), with minor heterogeneity (I^2^ = 2 %). Some patients had significant hip pain after arthroplasty; two studies assessed the pain rate after BHA compared with that after THA, and reported a significantly lower pain rate after THA (RR = 1.44; 95 % CI 1.02–2.00; *p* = 0.03; Fig. 12), but there was obvious heterogeneity between these studies (I^2^ = 88 %) [12, 27]. Significant difference was found in the operating time for BHA compared with BHA in four trials, (MD −14.83; 95 % CI −28.11, −1.55; *p* = 0.03; Fig. 13), with obvious heterogeneity (I^2^ = 91 %) [12, 28, 30, 31]. A significant difference was found in the blood loss between the two procedures in one report (MD −140.00; 95 % CI −221.01, −58.99; *p* = 0.0007; Fig. 14) [30]. No significant difference was found in the length of hospital stay in three trials [19, 29, 31] (MD −0.51; 95 % CI −1.87, 0.86; *p* = 0.47; Fig. 15), and there was no substantial heterogeneity (I^2^ = 0 %).

### Publication bias

**Fig. 16:**
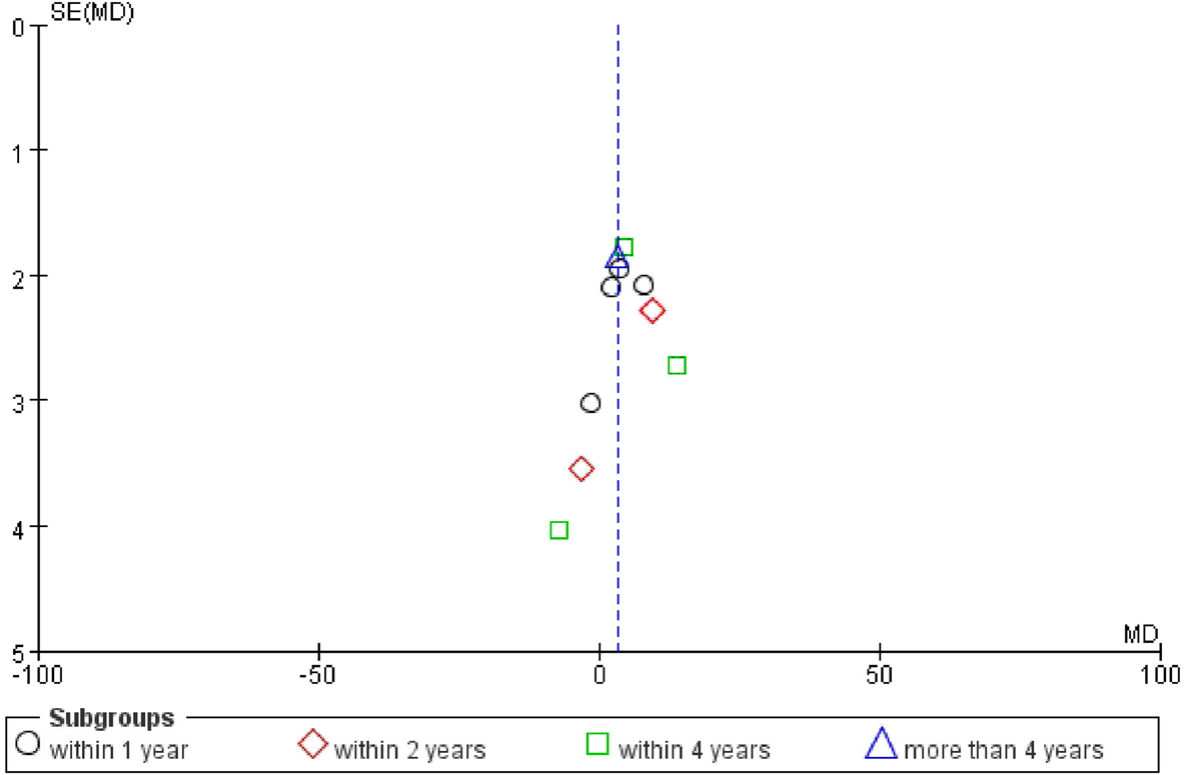
Funnel plot of publication bias in terms of Harris hip scores after bipolar hemiarthroplasty compared with that after total hip arthroplasty

No evidence of publication bias was found, and funnel plots were constructed for the HHS scores (Fig. 16). Although the funnel plots did not show substantial asymmetry, the impact of a possible publication bias cannot be excluded as the reliability of this kind of assessment is weak when a low number of studies are included.

## Discussion

The latest meta-analysis comparing THA with HA found that THA was preferable for healthy elderly patients with displaced FNF, because of better functional outcomes and lower reoperation rates [32]. However, in the last several years some results have suggested that BHA results in a better clinical outcome than unipolar HA [14, 15, 17], and that BHA should be the preferred treatment for elderly patients with displaced FNF [16]. There is still controversy over whether THA or BHA should be recommended in healthy elderly patients with displaced FNF.

Eight RCTs were included in the present analysis (total 1,014 patients; 523 with BHA versus 491 with THA). The different follow-up durations in the included studies meant that the data could not be directly pooled; thus the data were initially divided into four different subgroups (within 1 year, within 2 years, within 4 years, and more than 4 years). The results showed that the comparison of HHS was not different between BHA and THA in all subgroups, although HHS tended to be higher after THA. Both reoperation rate and acetabular erosion rates were higher after BHA after more than 4 years, while there was a higher risk of dislocation after THA. Some of the outcomes such as EQ_index_-5D, mobility and pain rate were in favor of THA, while operating time was in favor of BHA. No significant differences were found in other outcomes, including infection rate, general complication, 1-year mortality, blood loss, and length of postoperative hospital stay.

The present meta-analysis showed that HHS after THA was not significantly different from that after BHA in each subgroup; however, it tended to be higher after THA. It was reported in the last several meta-analyses that the HHS was higher after THA compared with HA [32–35]. The present meta-analysis included a newly reported high quality RCT in which the HHS after BHA was compared with that after polycarbonate–urethane (PCU)-THA within 3 years [31]. The PCU-THA comprised a PCU acetabular component coupled with a large-diameter metal femoral head for the treatment of displaced FNFs in elderly patients [31]. There were no significant differences between THA and BHA; however, the HHS tended to be higher in the BHA group than in the THA group within 3 years, and the authors suggested that the patients in the PCU-THA group experienced significantly more pain than the HA patients [31], which is one of the important factors affecting HHS [36]. These results might have influenced the pooled results in the present meta-analysis; therefore, we performed a sensitivity analysis by omitting this report. The subsequent results showed that the HHS in the THA group was better than that in the BHA group within 2 years, while the HHS was non-significantly higher in the THA group after 2 years. We suggest that, even though THA might lead to better clinical outcomes, proper implants were of great importance for the patients. Moreover, heterogeneity was obvious between studies, which might also have influenced the validity of the pooled results.

In the present meta-analysis, there were no significant differences between BHA and THA regarding reoperation rate within 4 years; however, the reoperation rate was higher after BHA than that after THA after 4 years. This result is similar to previous results [32, 33, 35, 37, 38]; however, it was different from the results of Kannan et al. [16]. Kannan et al. analyzed national registry data and reported a lower reoperation rate after BHA than after THA after a follow-up of 9 years in the Australian and Italian registries [16]. They concluded that the difference in reoperation rate after both procedures in Australia was not significant for patients under 75 years, but was significantly lower after BHA for those over 75 years and hence BHA may be better for this age group, considering the low functional demand and the morbidity of reoperation surgery in patients over 75 years of age [16].

There was a higher acetabular erosion risk after BHA than after THA, which often results in persistent pain [39–41] and reoperation after HA [42, 43]; this was even higher in unipolar HA [14, 15]. HA may lead to conversion from HA to THA, and Coates and Armour advocated that THA should be used in cases of acetabular erosion with HA [44], while Kannan et al. suggested that using THA to prevent acetabular erosion is not supported by registry data [16], although a longer follow-up may change this recommendation. However, the reoperation rate for acetabular erosion after HA was very low [16, 45], and accurate and careful preoperative planning for HA to equalize limb lengths and restore the patient’s own femoral offset can reduce postoperative acetabular erosion and pain [46]. Sen et al. introduced a new technique that was able to salvage a painful HA due to acetabular erosion [40].

One of the main reasons for reoperation after THA is dislocation [12]. In the present meta-analysis there was a higher dislocation risk after THA than after BHA, which was similar to previous reports [32–35, 37, 38]. Factors contributing to dislocation may include the surgical approach and the size of the prosthetic head. A multivariable analysis of dislocation after primary THA for all diagnoses found that a posterolateral approach and a smaller prosthetic head were associated with a higher rate of dislocation [47]. This was similar in BHA [16], hence choosing optimal hardware and improving the surgical access route may help to reduce complications associated with this common procedure. Enocson et al. analyzed the possible reasons related to dislocation in THA, and recommended the anterolateral approach for THA in patients with FNF to minimize the risk of dislocation [48]. Byström et al. studied the risk factors for prosthesis luxation leading to reoperation, and found that larger head size had been associated with fewer dislocations [49]. Therefore, an accurate and careful preoperative plan that considers the most appropriate approach and prosthesis may help lower the dislocation rate.

Our review has some limitations. First, the search was restricted to RCTs published in peer-reviewed journals, excluding other sources of biomedical literature that could have possibly provided more relevant studies. In such a case, studies with positive or statistically significant results would be expected to be over-represented in our review, as such studies were more likely to be published, particularly in the English language. To counter this, we used funnel plots to investigate the potential influence of publication bias on our results. Second, the validity of our results is limited by the low quality of the included studies; double-blinding was unattainable for most of the trials, which may decrease the strength of our conclusions. Third, there is the potential for bias because of high heterogeneity (≥50 %) in some comparisons, which may have affected the pooled results. Studies brought together in a meta-analysis will inevitability differ, and any kind of variability among studies may be termed heterogeneity. The included studies had clinical heterogeneity caused by variability in the participants (age, gender, comorbidities, preoperative ambulatory status), interventions (instrumentation from different manufacturers, different surgeons) and outcomes (selective reporting, data deficiency), and methodological heterogeneity caused by variability in study design and RoB.

However, there were several improvements in the present meta-analysis compared with previous reviews. This review is the most current report on the topic and includes the latest published trials. We adopted more strict inclusion criteria, with quasi-RCTs and non-RCTs strictly excluded in order to guarantee the reliability of results. We pooled most data of comparable parameters using subgroup analysis with different follow-up durations in an attempt to reduce the bias. We conducted this first analysis comparing BHA with THA to provide alternative guidelines for the clinical treatment of healthy elderly patients with FNF.

## Conclusions

For the healthy elderly with displaced FNFs, BHA led to better outcomes regarding dislocation rate, while THA was more favorable regarding acetabular erosion rate and reoperation rate. There were no significant differences between BHA and THA in some other important outcomes such as HHS, infection rate, general complications, and 1-year mortality. Further high-quality RCTs are needed to provide robust evidence and evaluate the treatment options for displaced FNF in healthy elderly patients.

## Abbreviations

BHA: Bipolar hemiarthroplasty
CBM: China Biological Medicine Database
CENTRAL: Cochrane Central Register of Controlled Trials
CI: Confidence interval
FNF: Femoral neck fracture
HA: Hemiarthroplasty
HHS: Harris hip score
ICTRP: International Clinical Trials Registry Platform
PCU: Polycarbonate–urethane
RCT: Randomized controlled trial
RoB: Risk of bias
RR: Risk ratio
THA: Total hip arthroplasty
